# Retraction: IL‐17A‐producing T cells exacerbate fine particulate matter‐induced lung inflammation and fibrosis by inhibiting PI3K/Akt/mTOR‐mediated autophagy

**DOI:** 10.1111/jcmm.18222

**Published:** 2024-03-20

Lu‐Hong Cong, Tao Li, Hui Wang, Yi‐Na Wu, Shu‐Peng Wang, Yu‐Yue Zhao, Guo‐Qiang Zhang and Jun Duan. IL‐17A‐producing T cells exacerbate fine particulate matter‐induced lung inflammation and fibrosis by inhibiting PI3K/Akt/mTOR‐mediated autophagy. *J Cell Mol Med*. 2020; 24: 8532–8544. https://doi.org/10.1111/jcmm.15475.

The above article, published online on 9 July 2020 in Wiley Online Library (wileyonlinelibrary.com), has been retracted by agreement between the authors, the journal Editor‐in‐Chief, Stefan Constantinescu, The Foundation for Cellular and Molecular Medicine and John Wiley and Sons Ltd. The authors reached out to the editorial office and asked to retract the article due to unreproducible results. Subsequent experiments did not confirm that PM2.5 can regulate PI3K/Akt/mTOR pathway to inhibit autophagy and promote inflammatory response and fibrosis of lung injury by inducing secretion of IL‐17A in γδT+/Th17 cells. Although the authors asked for the retraction, when asked to approve the retraction wording, they remained unresponsive.

